# Application of Behavioral Theories to Disaster and Emergency Health Preparedness: A Systematic Review

**DOI:** 10.1371/currents.dis.31a8995ced321301466db400f1357829

**Published:** 2015-07-01

**Authors:** Luche Tadesse Ejeta, Ali Ardalan, Douglas Paton

**Affiliations:** Department of Disaster & Emergency Health, School of Public Health, Tehran University of Medical Sciences-International Campus, Tehran, Iran; Private Consultant, Addis Ababa, Ethiopia; Department of Disaster Public Health, School of Public Health, Tehran University of Medical Science, Tehran, Iran; Department of Disaster and Emergency Health, National Institute of Health Research, Tehran University of Medical Science, Tehran, Iran; Harvard Humanitarian Initiative, Harvard University, Cambridge, USA; School of Psychological and Clinical Sciences, Charles Darwin University, Darwin, Northern Territory, Australia

**Keywords:** Behavior, disaster, Emergency Health, Model, preparedness, Theory

## Abstract

Background: Preparedness for disasters and emergencies at individual, community and organizational levels could be more effective tools in mitigating (the growing incidence) of disaster risk and ameliorating their impacts. That is, to play more significant roles in disaster risk reduction (DRR). Preparedness efforts focus on changing human behaviors in ways that reduce people’s risk and increase their ability to cope with hazard consequences. While preparedness initiatives have used behavioral theories to facilitate DRR, many theories have been used and little is known about which behavioral theories are more commonly used, where they have been used, and why they have been preferred over alternative behavioral theories. Given that theories differ with respect to the variables used and the relationship between them, a systematic analysis is an essential first step to answering questions about the relative utility of theories and providing a more robust evidence base for preparedness components of DRR strategies. The goal of this systematic review was to search and summarize evidence by assessing the application of behavioral theories to disaster and emergency health preparedness across the world.

Methods: The protocol was prepared in which the study objectives, questions, inclusion and exclusion criteria, and sensitive search strategies were developed and pilot-tested at the beginning of the study. Using selected keywords, articles were searched mainly in PubMed, Scopus, Mosby’s Index (Nursing Index) and Safetylit databases. Articles were assessed based on their titles, abstracts, and their full texts. The data were extracted from selected articles and results were presented using qualitative and quantitative methods.

Results: In total, 2040 titles, 450 abstracts and 62 full texts of articles were assessed for eligibility criteria, whilst five articles were archived from other sources, and then finally, 33 articles were selected. The Health Belief Model (HBM), Extended Parallel Process Model (EPPM), Theory of Planned Behavior (TPB) and Social Cognitive Theories were most commonly applied to influenza (H1N1 and H5N1), floods, and earthquake hazards. Studies were predominantly conducted in USA (13 studies). In Asia, where the annual number of disasters and victims exceeds those in other continents, only three studies were identified. Overall, the main constructs of HBM (perceived susceptibility, severity, benefits, and barriers), EPPM (higher threat and higher efficacy), TPB (attitude and subjective norm), and the majority of the constructs utilized in Social Cognitive Theories were associated with preparedness for diverse hazards. However, while all the theories described above describe the relationships between constituent variables, with the exception of research on Social Cognitive Theories, few studies of other theories and models used path analysis to identify the interdependence relationships between the constructs described in the respective theories/models. Similarly, few identified how other mediating  variables could influence disaster and emergency preparedness.

Conclusions: The existing evidence on the application of behavioral theories and models to disaster and emergency preparedness is chiefly from developed countries. This raises issues regarding their utility in countries, particularly in Asisa and the Middle East, where cultural characteristics are very different to those prevailing in the Western countries in which theories have been developed and tested. The theories and models discussed here have been applied predominantly to disease outbreaks and natural hazards, and information on their utility as guides to preparedness for man-made hazards is lacking. Hence, future studies related to behavioral theories and models addressing preparedness need to target developing countries where disaster risk  and the consequent need for preparedness is high. A need for additional work on demonstrating the relationships of variables and constructs, including more clearly articulating roles for mediating effects was also identified in this analysis.

## INTRODUCTION

According to the Center for Research on the Epidemiology of Disasters (CRED), natural disasters are classified as geophysical, metrological, hydrological, climatological and biological. These five disaster types encompass 12 disaster types and more than 30 sub-types. The 20th Century witnessed an increase in disaster losses, and this has continued its upward trend in the current Century. Climate change will increase the rate of increase of disasters, particularly those of meteorological origin. This is reflected in the fact that, of all natural hazards, floods are the most frequent and their impacts are also increasing 1 ^,^ 2. Though man-made disasters are also on the rise, the available global data is very limited to showing the trend over time; however, for the year 2012, CRED reported the occurrence of 188 technological disasters worldwide 1 **. **It is also important to note that geographically, Asia is the continent with highest toll of natural disasters (e.g., in 2012, it accounted for 40.7% of disasters and 64.5% of disaster victims). Discrepancies in between number of events and victims, with Asia bearing the brunt of both events and losses, highlights the urgent need for more DRR efforts to be directed to Asian countries. It is also evident that despite the growing experience of hazard events, the growing incidence of disaster related losses indicates that the experience of disaster per se is not acting to trigger greater mitigation or preparedness activities in at-risk populations. While it is generally agreed that preparedness at an individual, community and organizational levels is important for ameliorating hazard impacts, the fact that growing incidence of and awareness of disasters is not driving increased preparedness directly suggests that pursuing the goal of increased preparedness in practice is an activity that needs guidance. In developed countries this guidance has emerged in the forms of behavior change theories that have been used to model preparedness. While a major pandemic has not occurred in recent decades, the occurrence of SARS, bird flu, swine flu, MERS and Ebola, for example, has drawn attention to a need for more community preparedness for large-scale public health and epidemic hazards. The management of all of these diseases includes a behavior change component. Since there is likely to be, at least at a national level in most countries, overlap of responsibility for managing natural hazards, health hazards and technological hazards, it becomes important to gain a better understanding of the utility of behavior change theories to contribute across a range of risk management domains.

This argument is an extension of the need to provide empirical support for the all-hazards capability of a theory. Many places around the world face threats from several hazards (e.g., locations in Japan and New Zealand need to be ready to deal with the consequences of earthquake, tsunami, typhoon and volcanic hazards) and places in California need to prepare for earthquake and wildfire hazards. Consequently, it is important to ascertain whether a given theory can predict preparedness for hazards that differ in the type of preparedness required. All hazards require people to undertake survival actions (e.g., storing food and water), but they can differ in their respective structural and planning requirements (e.g., the need to secure a house to its foundations for earthquakes versus a need to create defensible space zones around a property for wildfire). The utility of a theory is thus a function of its ability to predict a range of behavioral outcomes.

Disaster preparedness is one of the basic components of DRR. Preparedness identifies the steps necessary to increase the likelihood of avoiding or minimizing hazard effect consequences. Preparedness strategies are developed through a hazard identification and mapping, vulnerability analysis and risk assessment 3 ^,^ 4, with behavior change strategies being used to inform how the outcome of this process can translate into protective actions. Effective preparedness reduces vulnerability, increases mitigation level, enables timely and effective response to a disaster event and so shortens the recovery period from a disaster, and increases community resilience 3.

Disaster and emergency preparedness efforts focus predominantly on human behaviors. Human behaviors derive from diverse factors that range from people’s risk perception to lessons from direct and indirect past experiences of disaster events and emergencies through to interaction between individuals and environment. These factors interact to influence the nature and level of people’s disaster and emergency health preparedness level 5 ^,^ 6 ^,^ 7 ^,^ 8 ^,^ 9. It is also clear from these studies that people within a given area, and who thus generally face comparable levels of risk, differ with regard to the nature and level of their preparedness and how people make choices about how to manage that risk. These efforts focus on how past experiences can be encapsulated in variables whose influence on behavior can be empirically tested. For example, people’s experience in successfully dealing with challenging events in the past can be captured using scores on a measure of self-efficacy.

Hence, if variables can be consistently implicated as components of behavioral change interventions, in different parts of the world, this knowledge can be used to inform education programs that aim to deliver messages informing and educating people about protective measures. Research has found such approaches can effectively facilitate disaster preparedness behaviors 10 ^,^ 11. However, the evidence is not unequivocal. For example, some studies have found that giving information or education to the community doesn’t necessarily lead to disaster and emergency preparedness, and how the risk itself would be interpreted by individuals could partially determine the process and the level of disaster and emergency preparedness at a given time 12 . The question then becomes one of asking why such contradictory findings have occurred.

One reason relates to identifying the cognitive, affective, emotional and other social relationship and interaction factors that influence how individuals’ interpret environmental risk information and how this relates to behavioral preparedness for disaster and emergency. That is, one reason for the discrepancy in the effectiveness of information-based change programs discussed above relates to whether agencies used an evidence-based approach or a more ad hoc approach to their risk communication programs. It is also possible to hypothesis that, amongst the former, agencies differed in the theory adopted to inform their program design. Given the existence of numerous theories that describe the interpretive processes that inform behavior change, identifying salient variables and behavior change processes becomes a challenging task. Investigating this, however, is important from the perspective of giving risk management policy makers and planners the processes they need to guide how to develop and combine information and behavior into the required interventions.

Though there have been several initiatives that have applied behavioral theories to disaster and emergency preparedness little is known as to which behavioral theories are more commonly used, where they have been used, and why any one theory has been preferred over other behavioral theories. Therefore, as there is no previous systematic review conducted, this study has been intended to search evidence to get answers to these questions by assessing the application of behavioral theories to disaster and emergency preparedness across the world. The major objectives of this systematic review were to: (i) identify which behavioral theories have been applied to disasters and emergency health preparedness and investigate why these theories were preferred over others; (ii) assess as to which theories have been applied with regard to specific natural and man-made disasters and emergencies preparedness; (iii) examine the most common theories and models applied in different regions of the world pertaining to various natural and man-made disasters and emergency preparedness; and (iv) investigate and analyze the methods of analysis used for each study of disaster and emergency health preparedness.

## METHODS

**Study design**

In order to assess the application of behavioral theories to disaster and emergency health preparedness, this study used a systematic literature review. The systematic literature review protocol was prepared to guide the development of the study objectives, questions, inclusion and exclusion criteria, and search strategies were developed and pilot-tested at the beginning of the study. However, the protocol was not registered.

**Research questions**

The following research questions were established for the systematic review during the preparation of the study protocol: (i) what are the most commonly applied behavioral theories to disaster and emergency health preparedness across the world?; (ii) what are the major reasons of choosing one theory over the other specific to each disaster and emergency preparedness?; (iii) in which part of the world are the behavioral theories widely applied to disaster and emergency health preparedness?; (iv) what are the challenges in applying the behavioral theories to disaster and emergency health preparedness?; (v) are studies applying behavioral theories to disaster and emergency health preparedness more common in areas where the rates of disaster events are widespread? ; and (vi) are the methods of analyses used for each behavioral theory applied to disaster and emergency health appropriate in explaining the relationships of constructs within each theory or model?

**Inclusion and exclusion criteria**

The inclusion criteria used for this systematic review were: studies applying behavioral theories to all disasters and emergencies preparedness; studies conducted in all regions across the world; original articles that have been published in peer reviewed journals; and studies conducted on any disease epidemic preparedness applying behavioral theories or models. The references of review papers related to disaster and emergency preparedness were also referred to, so as to archive the relevant articles. There was no restriction applied to the date of studies sampled, and all databases were searched for studies conducted till the last date of our search (November 12, 2014). On the other hand, studies applying behavioral theories and models to response and recovery phases of disaster management, conference papers, non-English language articles, and disaster preparedness studies conducted without applying the behavioral theories and models were the exclusion criteria.

**Information sources and search strategies**

The sources of information for our systematic review were PubMed, Scopus, Mosby’s Index (Nursing Index), and Safetylit databases. Apart from these databases, the relevant articles were searched in Google scholar and other sources to archive relevant documents. The search key terms were framed along three major categories: behavioral theories; disasters and health emergencies (natural, man-made disasters and emergencies), and preparedness. See table 1 for the detail search strategies.

**Table 1: Information sources and search strategies:** 

Database	Keywords	Search outcome (number of articles obtained)	Last date of search
PubMed	((theor* [tiab] OR model* [tiab] AND behavior*[tiab]) OR “health belief model” OR “theory of planned behavior” OR “social cognitive theory” OR “trans theoretical model”) AND (disaster*[tiab] OR emergency* [tiab] OR storm* [tiab] OR cyclone* [tiab] OR typhoon*[tiab] OR hurricane*[tiab] OR tornado*[tiab] OR drought*[tiab] OR earthquake*[tiab] OR flood*[tiab] OR tsunami*[tiab] OR volcano*[tiab] OR “chemical terrorism” OR “biological terrorism” OR “agro terrorism” OR “nuclear terrorism” OR epidemic*[tiab] OR outbreak*[tiab] OR pandem*[tiab]) AND (prepar*[tiab] OR read*[tiab] OR “protective action” OR “adaptive behavior” OR plann*[tiab])	229	November 12, 2014; 10:30 AM
Scopus	TITLE-ABS-KEY(theor* OR model* AND Behavior*) OR (“health belief model” OR “theory of planned behavior” OR “social cognitive theory” OR “social network and social supports” OR “trans theoretical model”) AND TITLE-ABS-KEY (disaster* OR emergency* OR storm* OR cyclone OR typhoon* OR hurricane* OR tornado* OR drought* OR earthquake* OR flood* OR tsunami* OR volcano* OR “chemical terrorism” OR “biological terrorism” OR “agro terrorism” OR “nuclear terrorism” OR epidemic*) AND TITLE-ABS-KEY (prepar* OR read* OR “protective action” OR “adaptive behavior” OR plann*). [English language] marked	1,672	November 12, 2014; 10:15 AM
Mosby’s Index(Nursing Index)	(theory OR model) AND (behavior OR “health belief model” OR “theory of planned behavior” OR “social cognitive theory” OR “social network and social supports” OR “trans theoretical model”) AND (disaster OR emergency OR storm OR cyclone OR typhoon OR hurricane OR tornado OR drought OR earthquake OR flood OR tsunami OR volcano OR “chemical terrorism” OR “biological terrorism” OR “agro terrorism” OR “nuclear terrorism” OR epidemic OR outbreak OR pandemic) AND (preparedness OR readiness OR “protective action” OR “adaptive behavior” OR plan)	95	November 12, 2014; 12:30 PM
Safetylit	(theory OR model) AND (behavior OR belief OR perception) AND (disaster OR emergency OR hazard) AND (preparedness OR readiness OR plan)	44	November 12, 2014;11:30 AM

**Study selection, data extraction and analysis**

First, articles were assessed based on their titles and then on the basis of abstracts in order to exclude articles not fulfilling the inclusion criteria. The remaining articles’ full texts were archived and read, and at this stage, articles that did not meet the inclusion criteria were rejected. In order to search for themes of selected articles and to categorize and extract data for analysis, the abstracts of selected articles were formatted on Microsoft Word and changed to text files. The text files of abstracts were then imported to open code version 4.0 qualitative software 13. Themes were carefully selected from each imported abstract and coded, followed by synthesis at two levels (Synthesis 1 and Synthesis 2). The synthesized data were used for categorizing the articles into different themes and data were extracted from each full-article according to the themes. The main extracted data were analytical part of each study’s results, which were related to constructs of each theory or model. The assessment for risk of bias at each study was done by critically appraising the methods of data collection, and also the type of statistical analysis and its implication on the outcome of each of the study assessed. The results were presented using qualitative and quantitative methods. The focus of the analysis was on the main constructs of each theory, and to save space sometimes the results were presented in qualitative terms. This systematic review used the “PRISMA 2009 Checklist” as a guide wherever possible (Appendix-1).

## RESULTS

From all databases, a total of 2040 titles, 450 abstracts and 62 full texts of articles were assessed for eligibility criteria, whilst five articles were archived from Google Scholar and other sources. Finally, 33 articles fulfilling the inclusion criteria were selected to be reviewed (See Figure 1). As one of the selection criteria, these 33 articles were selected for using different behavioral theories and models to study the disaster and emergency preparedness status for various hazards across the world. Using the open code software, the 33 articles were categorized as: disease outbreak preparedness (14 articles), flood disaster preparedness (6 articles), earthquake preparedness (3 articles), preparedness for climate change including heatwaves (3 articles), tornado preparedness (2 article), terrorism preparedness (3 articles), general emergency preparedness (2 articles), and one article deals with the disaster preparedness for both flood and earthquake and one article was about earthquake and tornado preparedness. For each category, the results of selected articles were presented.

**Article Selection Flow Chart:**
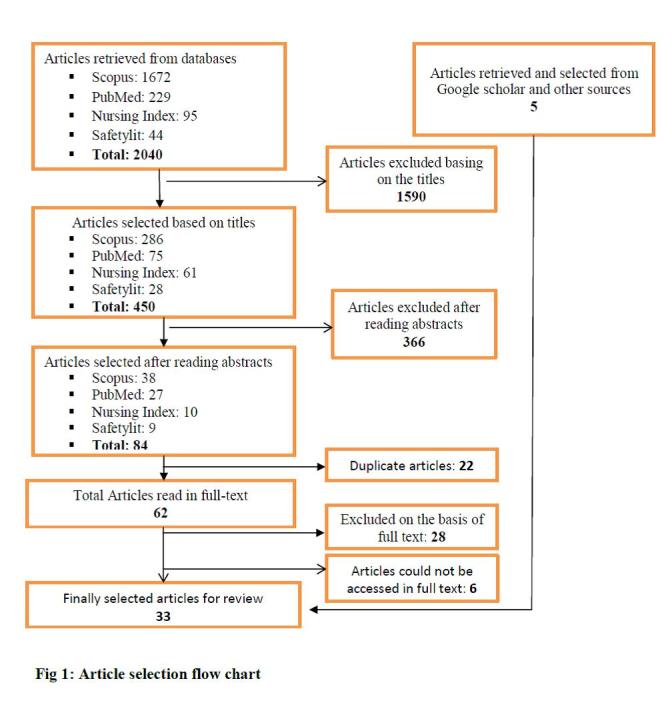

**Disease outbreak preparedness**

We found 14 studies that had been conducted on disease outbreak preparedness using different behavioral theories and models. Of these, five studies were on vaccination against A/H1N1 influenza (swine flu), using the Health Belief Model (HBM) 14 ^,^ 15 ^,^ 16, the Theory of Planned Behavior (TPB) 16 ^,^ 17 , and the Multidimensional Locus of Control (MLOC) Theory 18. Four of the articles archived assessed health workers preparedness for emergency response, applying Witte’s Extended Parallel Process Model (EPPM) 19 ^,^ 20 ^,^ 21 and the TPB 22. During the full text review phase of the selected studies, two articles on blood donors’ intention to donate blood during the low and high risk of influenza outbreak were identified, however, it was recognized that the same study was published in two different journals 23 ^,^ 24. Hence, the analysis of results for this particular case was based on only one article; that used the TPB to investigate the beliefs of blood donors during the low and high risk phases of avian influenza outbreak 23. Moreover, the EPPM was applied to assess preventive measures under taken against H5N1 25; Protective Motivation Theory (PMT) was used to investigate non-pharmaceutical measures against unspecified influenza 26; Social Cognitive Model was applied to assess preparedness for swine flu 27; and Social Predictor Model of Intentions (SPMI), a new model derived from previously existing models, was used to assess the preparedness for a pandemic flu 28.

The major reason cited regarding preference in studies that applied the HBM to disease outbreak preparedness, was the HBM’s history empirically predicting preventive health behavior 14 ^,^ 15 ^,^ 16 ^,^ 18. However, it was integrated with other models or theories in three studies 16 ^,^ 17 ^,^ 18. One study stated that HBM had more predictive power of behavior than explaining behavior 16. The rationale for use of TPB for disease outbreak preparedness include TPB’s explanatory power along with social cognitive theory 16, TPB’s widely applicability to predict the intention and behavior 17 ^,^ 23, and TPB’s more appropriateness “for situations where individuals do not perceive themselves as having complete control over their behavior” 22. The MLOC theory was applied, as stated by one study, due to the MLOC’s capability to explain the behavior of individual and its applicability in situations whereby hazards may be beyond the control of people. In doing so, however, it was used along with the HBM to test and for the purpose of comparison 18. Four studies applied the EPPM to assess preparedness in the context of disease outbreaks, and the rationale for its application were the “usefulness” of EPPM to understand adaptive behavior “in the face of unknown risk” 19, EPPM’s “usefulness” to understand “how health care may positively or negatively influence their [health workers’] willingness to fulfill the response expectations” 21, and to test the model 25. One study that applied EPPM not clearly stated the reason of using it 20. PMT was applied to further build on the previous studies 26, while the Social Cognitive Model was used to test the model on behavioral responses to pandemic influenza 27, and SPMI derived from previous theories and models to test the relationship between personal, community, social factors and the formation of intention to act, and the mediation of social trust 28.

Based on the cross-sectional studies that applied HBM, perceived susceptibility 14 ^,^ 16 ^,^ 17 , perceived severity 14 ^,^ 17 ^,^ 18 , perceived benefits 14 ^,^ 16 ^,^ 18 , and perceived barriers 14 ^,^ 16 ^,^ 18 consistently and significantly predicted the vaccination status or the intention to get vaccinated (See table 2).One study that applied the HBM to investigate factors influencing the vaccination status of pregnant women didn’t clearly report the components of HBM positively or negatively determining the vaccination status 15. The study populations of these five studies were less than 40 years of age (n =387) 14, pregnant women (n=250) 15 , aged 19 to 24 years (n =473) 16, older than 18 years (n= 134) 17, and 65 years or older (n = 2147) 18. Thus, the results of each of these studies cannot be generalized to other age groups of their respective general populations, and the small sample size 17 present in one study could threaten its internal validity. In addition, these studies were conducted at higher institutes 14 ^,^ 16, hospital 15 and online 17; and hence the studies’ settings could limit generalizing the results to the same populations who are not in a higher institute, not regularly visiting hospital during pregnancy, and who are not regular (or who do not use at all ) internet users, respectively. The response rates of three studies were quite high, ranging from 88.8% to 94.8% 14 ^,^ 15 ^,^ 16, while one study sent a questionnaire two times and the average response rate was 58% 18, and in one study the response rate was not reported 17. Therefore, the interpretation of the results of each of these studies needs to take into account sample size, setting and response rate; as these factors among others introduce potential sources of bias into the nature and interpretation of the respective studies’ findings.

**Table 2: HBM and vaccination status/intention to receive the vaccine of swine-flu/influenza-virus:** OR=Odds Ratio; CI=Confidence Interval; P-values considered significant at <0.05; **-** result not provided

Constructs of HBM			Teitler-Regev S, et al. 14			Taylor P, et al. 16			Nexøe J, et al. 18
Constructs of HBM	OR	95% CI	P-value	OR	95% CI	P-value	OR	95% CI	P-value
Susceptibility	0.23	-	0.01	0.13	-	< 0.05	-	-	-
Severity	0.18	-	0.01	0.05	-	>0.05	1.11	1.07-1.15	0.000
Risk	0.47	-	0.04	-	-	-	-	-	-
Self-efficacy	-	-	-	0.07	-	>0.05	-	-	-
Benefits	0.7	-	0.43	0.27	-	<0.001	1.64	1.31-2.03	0.000
Barrier	0.32	-	0.01	0.04	-	>0.05	0.89	0.85-0.94	0.000
Cues to action (Health promotion)	1.3	-	0.55	-	-	-	-	-	-

Three cross-sectional studies used the EPPM to assess mainly the willingness of public health employees (n =1835, with response rate 83%) 19, local public health department and hospital workers (n= 3426, with response rate 66%) 20, and hospital workers only (n=3426, response rate 18.4%) 21 to respond to an emergency during pandemic influenza. The EPPM relies on two basic components, perceived threat and perceived efficacy. In three of the studies that applied this model, it was shown that perceived higher threat and higher efficacy predicted the willingness of health workers to respond to an emergency when either required or asked 19 ^,^ 20 ^,^ 21. Moreover, these studies found perceived efficacy to be a more significant (i.e., higher predictive utility) component of the EPPM in determining the health workers’ willingness to respond to pandemic influenza/flu emergency 19 ^,^ 21. In addition, perceived psychological preparedness reported to be a vital predictor of willingness to respond to emergency 19 ^,^ 20 ^,^ 21 (refer table 3). As the aims of these studies were to determine the willingness of health workers to respond to emergency, the set-up and sampling method used are robust, and the results could be generalized to beyond the study populations.

**Table 3: Associations between EPPM categories and self-reported willingness to respond to emergency:** OR= Odds Ratio (adjusted for demographic characteristics ^19,21^); CI= Confidence Interval

Willingness to respond	Low Threat, High Efficacy	High Threat, Low Efficacy	High Threat, High Efficacy
Willingness to respond	OR (95% CI)	OR (95% CI)	OR (95% CI)
Barnett DJ et al. 19			
If required	16.48 (5.16–52.65)	2.39 (1.48–3.87)	41.58 (10.15–170.40)
If asked but not required	5.31 (2.93–9.61)	1.43 (1.00–2.04)	8.46 (4.77–15.01)
Balicer RD et al. 21			
If required	13.09 (7.67 - 22.34)	1.41 (1.05 - 1.90)	9.25 (5.94 - 14.40)
If asked but not required	7.12 (4.94 - 10.25)	1.10 (0.85 - 1.42)	5.52 (4.03 - 7.56)

Another study that applied the EPPM among population aged 18- 24 years (n= 265) to test the utility of the Model in relation to use a fear message based intervention in relation to preparedness for the H5N1 virus demonstrated (using structural equation analysis) the existence of a positive association between perceived threat and fear arousal (standard regression coefficient (b) = 0.55, p < 0.5), fear arousal and behavioral intention (b=0.23, p< 0.051), perceived efficacy and behavioral intention (b=0.47, p < 0.05) 25. However, this study used a relatively small sample size which could be one of the limitations and the response rate was not reported so as to critically assess the strength of evidence generated from the study. The latter represents a significant limitations (the response rate was not reported) with regard to critically assessing the strength of evidence generated from the study and calls for its conclusions to be regarded as tentative until further work is conducted.

The TPB was applied to assess the intention of getting swine flu vaccine in the face of the epidemic 16 ^,^ 17 , and to investigate nurses’ intention and volunteering to care for Severe Acute Respiratory Syndrome (SARS) patients (n= 750, response rate 90%) 22, and to study blood donors’ intention to donate during the influenza epidemic among aged 16-72 years population (n=172) 23. As its major constructs, TPB has three components that include attitude, subjective norm and self-efficacy (perceived behavioral control). Based on “hierarchical ordinary least square regression” analysis, a study conducted in the USA reported that attitude (b=0.25, p<0.001) and subjective norm (b=0.40, p<0.001) were positively associated with behavioral intention for swine flu vaccination; whereas self-efficacy was not associated with behavioral intention (b=-0.7, p>0.05) 16. A similar pattern was observed in a study conducted in UK (analysed using “Hierarchical ordinary least square regression”), that reported that attitude (b= -0.51, p < 0.001) and subjective norm (b= - 0.20, p < 0.01) inversely predicted behavioral intention for swine flu vaccination; while self-efficacy (b= - 0.08, p > 0.05) did not predict behavioral intention 17. With regard to predicting nurses intentions to care for SARS patients, a path analytical study identified, attitude (b=0.25, p< 0.001) and self-efficacy (b= 0.39, p<0.001) as predictors. However, based on our own interpretation of the findings reported, the variable subjective norm was not included in the path analysis in this study 22. From a study investigating blood donors’ intention to donate during high and low risk periods of avian influenza, clear evidence was not generated, as the major constructs of TPB were not clearly analyzed and summarized in a way that permitted the association of intention with those variables; sample size of the study was small and response rate wasn’t reported 23.

The predictive power of the Multidimensional Locus of Control (MLOC) theory has been tested in the context of influenza vaccination. It doing so, it has been compared with the HBM. The MLOC theory comprises three major constructs, “internal locus of control”, “powerful others locus of control”, and “chance locus of control”. It was reported that MLOC theory did predict influenza vaccination status. However, the MLOC theory was found to have less predictive power (Positive Predictive Value= 54%, 95% CI =45–62%; Negative predictive value = 66%, 95% CI = 63–69%), compared with the HBM (positive predictive power=76%, 95% CI =71–80%; and negative predictive power=82%, 95% CI =79–85%)^18^. One study used PMT to investigate non-pharmaceutical proactive measures against influenza outbreaks under mild and severe influenza scenario (n=443, response rate 59%, participation rate 44%). PMT includes threat appraisal and coping appraisal as its major elements. According to this particular study that applied PMT, threat appraisal was measured by perceived personal risk, emotional response to threat and perceived severity of health threat; on the other hand, coping appraisal was measured by general response efficacy, self-efficacy, and response costs. According to the results this study, coping appraisal was found to explain the self-reported intention of pharmaceutical measures 26.

A Social Cognitive Model using a cross-sectional study design, with a participant population comprising those 18 years and older, was applied to assess factors predicting the recommended behavior for pandemic influenza prevention (n =1010). The outcome of the analysis showed that affective response mediating the relationship between cognitive evaluations and social-contextual factors and compliance with the recommended behaviors; while coping efficacy and preparedness of institutions were not associated with the recommended behaviors 27. Another cross sectional study (n =400) that used a SPMI, reported (using structural equation model analysis) that negative outcome expectancy inversely related to intention (-0.12); positive outcome expectancy related to intention (0.37) and articulating problems (0.24) and community participation (0.18). In addition, articulating problems in turn related to empowerment (0.12); community participation related to empowerment (0.75), which in turn related to trust (0.66). Moreover, community participation inversely related to trust (-0.37) and related to intention (0.25); and in the end intention was shown to be related to preparation (0.16) 28.

**Flood disaster preparedness**

Seven studies were identified as addressing flood disaster preparedness, including one study that combined flood and earthquake hazards preparedness. As such, the Protective Action Decision Model (PADM) 29, affective and cognitive routes 30, expectancy-valence models (model of risk reduction process) 31, outcome expectancy and self-efficacy 32, Protective Motivation Theory (PMT) 33, Vested Interest Theory (VIT) 34, and the social cognitive model 35 were identified as theories and models that had been applied to assess flood hazard preparedness. PMT and VIT were chosen, as stated by their respective studies, as being adopted with the goal of improving on previous works by modifying or adding additional variables 33 ^,^ 34. The range of theories used to assess flood preparedness provided an opportunity to examine the diversity of variables and relationships between variables in behavior change theories and models. The PADM “has a more detailed set of salient beliefs” and its clarity in terms of “response costs” compared with PMT 29. The rationale for using the Expectancy-valence models (model of risk reduction process) derived from PMT, the PADM, and Paton’s social cognitive preparation model was to assess the risk reduction process in an all-hazards context 31. For example, the rationale for using the social cognitive model, which was initially developed to test volcanic hazards preparedness, was to test whether its predictive utility was sustained when applied to predicting earthquake and flood hazard preparedness 35. The other studies discussed here did not explicitly provide reasons for their selection of the theories/models they applied to flood disaster preparedness 30 ^,^ 32.

A cross sectional study that applied PADM (n=1115, with response rate 12.9% in coastal and 9.6% in river regions) reported that hazard related attributes and risk perception were positively associated with flood disaster preparedness intention, while resource related attributes were negatively associated with preparedness intentions 29. Another similar study (n =1071, response rate 11.8% in coast, 9.6%-12.9% in river areas) that set out to assess affective and cognitive routes to preparedness intention reported that both cognitive and affective routes influencing preparedness intentions. More specifically, higher level of trust associated with lower level of individuals’ perception about flood likelihood (b = -0.46, p < 0.001), which in turn reduced flood preparedness intentions (b = 0.46, p < 0.001). Furthermore, in the same study, trust influenced and reduced dread level induced by flood (b= -0.29, p< 0.001), which was associated with low level of preparedness intentions (b = 0.2, p < 0.05). Along with trust, emotions related to previous flood hazard experiences influenced the level of dread that could be revealed by individuals 30.

Findings from an expectancy-valence model study among 78 representatives from municipalities and associations and 11 citizens, revealed that risk appraisal, coping appraisal, and some components of institutional context variables correlated with risk reducing behavior 31. It should be noted that outcome expectancy and self-efficacy were shown to be correlated with intention for flood preparedness, as per the study conducted among 286 adult residents in the study population area 32. Whilst results from a study (n=157) that used PMT, concluded that threat experience appraisal, threat appraisal and coping appraisal interacted to predict four components of protective responses of flooding 33. According to findings from a VIT study (n=659), among individuals aged 20-69 years residing in higher flooding risk areas reported more experience of flooding, perceived higher risk, and had more concern. However, with regard to their preparedness intentions, no difference was observed between individuals residing in high and low risk areas of flooding 34. As per the results from the quantitative report of the study (n=264 cases for flood hazard) that used Paton’s social cognitive model, community participation, positive outcome expectancies, negative outcome expectancies, collective efficacy, general trust, and empowerment were directly or indirectly (being mediated by other variables) were able to significantly predict (tested using structural equation model analysis) people’s intentions to prepare for floods 35.

In general, studies pertaining to flood hazard preparedness targeted coastal and river areas. In two of these studies response rates were very low, ranging from 9.6% to 12.9% 29 ^,^ 30, while another study that sampled from the municipalities and associations produced a relatively acceptable rate of return (62.4%) 31, while in four studies, response rates were undisclosed 32 ^,^ 33 ^,^ 34 ^,^ 35. Low sample sizes are observed among two studies 31 ^,^ 33. Thus, selection bias is one of the major limitations of studies regarding flood hazard and disaster preparedness.

**Preparedness for earthquake**

Four studies addressing earthquake preparedness were identified. These studies applied the social cognitive model 35 ^,^ 36, the VIT 37 and the Person-relative-to-event model 38. The rationale presented for the selection of these models for researching earthquake preparedness derived from their earlier success in predicting preparedness for other hazards (e.g., volcanic preparedness) 35. A second goal of these studies was to build further on previous studies and so assess the all-hazards capabilities of each theory 36 ^,^ 37 ^,^ 38.

Similar to what has been reported above for the flood preparedness, the study (n=256 for earthquake hazard) that applied the social cognitive model to test for earthquake preparedness reported that all variables directly or indirectly contributed to predicting intentions to prepare for earthquakes. However, structural equation model for intentions to prepare for earthquakes accounted higher variance (37%) in comparison to intentions for floods preparedness (20%) 35. Another study that used social cognitive model made data collection and analysis as “phase-one” and “phase-two”. In phase one (n=660 with response rate 27.5%), it was reported that risk perception, critical awareness and earthquake anxiety (being mediated by outcome expectancy, self-efficacy and action coping) influenced intention to prepare. In addition, critical awareness directly and indirectly predicted intention to prepare and intention to seek information (with these being linked to predicting the adoption of protective measures and not adopting measures respectively). In phase two analysis of this study (n= 640 with response rate 27%), from all variables, only intention to prepare (mediated by time) was able to predict actual preparation for earthquake (b = 0.78) 36.

A cross-sectional and qualitative study (n=56 for earthquake hazard, with a response rate of 76%) applied the VIT to examine preparedness for earthquake and tornado hazards; however, this study did not generate any evidence that could support an association between VIT variables and preparedness 37. The “quasi-experimental” study (n= 240 with response rate 73%) that used the Person-relative-to-Event (PrE) model, discussed how persons with sufficiently high resources and with high perceived threat of earthquakes showed higher level of preparedness compared with persons with low resources and high threat of earthquake (t (25) = 2.21, p < .05); and persons with low resources and with low level of threat showed more preparedness than those with high resources and low level of threat (t (23) = 2.36, p <.05) 38.

The responses rates of the studies related to earthquake ranged from 27% to 76% 36 ^,^ 37 ^,^ 38 and the sample sizes were not as such large in all quantitative studies (ranging from 256 to 660 participants), and in the case of qualitative study, the sample size (n=56) taken was convincingly sufficient 38 and should not be judged as quantitative studies’ samples. On another strength point, the selection methods in all were random that could defend the findings obtained as non-bias. Moreover, unlike the other hazards which are dominated by cross sectional studies, from studies regarding earthquake preparedness there was one study with a design of “quasi-experimental” method 38, and evidence from this study could be more robust than cross-sectional studies.

**Preparedness for environmental hazards, climate change and heat waves**

Social-ecological resilience theory and the HBM have been applied to assessing the emergency preparedness for environmental hazards 39, climate change 40, and heat waves 41. The study that applied the HBM to assess the adaptive behaviors of individuals during a heat wave stated that this theory was selected as some of its constituent constructs “relate to perception” in line with the focus of the study 41, while the remaining two studies did not clearly specify the reason for selection of those theories 39 ^,^ 40.

The study (n=64) that used social-ecological resilience theory, reported that vulnerability (educational attainment) and adaptive capacity were associated with adoption of household emergency plans, but that exposure was not significantly associated with emergency planing. However, its small sample size and the purposive sampling employed may have biased the study results 39. Another study (n=771) that applied the HBM to climate change hazards, using logistic regression and path model analysis. The logistic regression analysis concluded that perceived barriers and perceived cues to action were positively and significantly associated with having an emergency kit and an emergency plan. Whereas, in the path analysis, perceived barriers (b=0.16, p < 0.001) positively associated with having an emergency plan; perceived susceptibility (b = 0.100, p < 0.01), perceived severity (b == 0.108, p < 0.01), perceived benefit (b = 0.108, p < 0.01), and perceived barriers (b = 0.213, p < 0.001) were associated with having an emergency kit 40.

A cross sectional study (n=490) that assessed the adaptive behaviors for heatwaves merged perceived susceptibility into perceived severity to form one construct, termed “risk-perception”. Using logistic regression analysis, and controlling for other variables, perceived benefit (OR = 2.14, 95% CI =1.00-4.58) and cues to action (OR = 3.71, 95% CI = 1.63-8.43) were significantly associated with adopting adaptive behaviors; whereas risk perception (OR = 0.66, 95% CI = 0.29-1.46) and perceived barriers (OR = 0.82, 95% CI = 0.31-2.13) did not significantly predict the adoption of adaptive behaviors 41.

**Preparedness for tornado**

Two studies were identified that focused on tornado hazards preparedness. One of these studies (n=487 with response rate 71%) applied the VIT, integrating tornado and earthquake preparedness as reported above 37.The second study (n=715) used a theory called “precaution adoption theory”. Precaution Adoption Theory (PAT) identifies absolute risk perception, relative risk perception, negative affect, fear, preoccupation and perceived control as predictors, and protective action as the dependent variable 42. As stated above, the study applying the VIT to tornado and earthquake did not produce any evidence supporting the applicability of this theory for preparedness behaviors for these hazards 37. After controlling for other variables, results from the PAT study demonstrated that preoccupation with tornadoes, recollections of fear and negative affect when thinking about tornadoes were associated with protective action; and absolute risk perception, relative risk perception, and perceived control couldn’t maintain statistical significance to predict protective action 42.

In the Introduction to this paper, the need for theories to be able to demonstrate all-hazards capability was discussed. The above discussion has focused on hazards (geological, meteorological, health) that are predominantly natural in origin. To be able to argue for all-hazards capability, preparedness theories also must demonstrate their utility for hazards of human origin that present people with different sources of risk (e.g., from deliberate human action versus natural causes or Acts of God). Increasingly prominent in this category is acts of terrorism.

**Preparedness for terrorism**

Three studies were identified that related to preparedness for terrorism. Three of them used less prominent models or theories compared with those discussed so far in any field of study. One study (n=3300, response rate 35%) adapted a model from PMT, focusing on risk perception and preparedness for terrorism. This study reported that risk perception did not directly predict preparedness for terrorism. The relationship between risk perception and preparedness of acts of terrorism was mediated by knowledge, perceived efficacy and milling behavior 43. The second study (n=3300, response rate) used a model called the “theory of communicating actionable risk.” This model designed mainly on information (observed, received-content and density) as determinant factors and preparedness actions as dependent variable; knowledge, perceived effectiveness of preparedness, milling behavior, were treated as mediating factors. This second study reported that information observed and received directly and also being mediated by the above variables predicted the preparedness actions 44. The third study (n=800), termed the model it applied for terrorism preparedness, “adaptation terror preparedness.” In this model, in addition to its incorporation of socio-demographic and past experience factors, also included risk perceptions, social networks, emotive, and information as the predictors of adaptive behavior for terrorism. The results of this third study revealed that apart from the socio-demographic factors, the other variables posited as being predictors did not act as predictive factors of adaptive behavior for terrorism 45. The first two studies 43 ^,^ 44 used data from the same study participants. In all three studies, the sample sizes were large and the sampling method was random, which supports the validity of the results reported.

This section opened with a reiteration of the need for theories to demonstrate all-hazards capability. The discussion of this topic has so far involved comparison across hazards. An alternative approach is to explore general emergency (as opposed to hazard specific) preparedness. This is the approach adopted in the studies discussed in the next section.

**General emergency preparedness**

Two articles were found addressing general emergency preparedness at household level and with regard to volunteers’ willingness to respond to various kinds of hazards. To build on the previous studies, one of the two studies (n=1302 with response rate 40.5%) applied the Trans-theoretical Model (TTM) to investigate the individuals’ emergency preparedness based on study participants’ acquisition of information from the media about any natural, man-made, and influenza related disasters and emergencies. This study reported that self-efficacy, subjective norm, and exposure to emergency news were positively associated with emergency preparedness and having emergency items 46. The second study (n=3181) too, set out to further the previously existing knowledge on emergency preparedness by applying EPPM to assess the willingness of volunteers to respond to disasters emanating from weather, pandemic influenza, radiological, and bio-terrorism hazards. Thus, according to the results of this second study, despite the variation of association of between self-efficacy and willingness to respond across the above listed hazards scenarios, self-efficacy was found to be sole predictor of willingness to respond; while other components of the EPPM were not statistical significantly associated with willingness to respond 47. Though the second study 47 did not specifically report the response rate of study participants, both studies’ sample sizes were large and both selected participants at random, reducing the risk of their results being affected by selection and information bias (standardized questionnaires were used in both cases).

## DISCUSSION

The protocol developed for this study identified 33 relevant articles fulfilling the criteria from four databases and other sources. Of these 33 studies, 13 were conducted in USA 16 ^,^ 19 ^,^ 20 ^,^ 21 ^,^ 37 ^,^ 38 ^,^ 39 ^,^ 40 ^,^ 42 ^,^ 43 ^,^ 44 ^,^ 46 ^,^ 47 and remainder from New Zealand and Australia combined (5 studies) 23 ^,^ 28 ^,^ 35 ^,^ 36 ^,^ 44 , the Netherlands (2 studies) 29 ^,^ 30, Germany (2 studies) 31 ^,^ 33, Italy (2 studies) 27 ^,^ 34, Israel (2 studies) 14 ^,^ 45, and one each in Canada 15, Sweden 26, Denmark 18, China 25, Taiwan 22 and India 32 (please refer to Appendix-2 for details). The most widely applied theories and models from the identified studies were the HBM (6 studies) 14 ^,^ 15 ^,^ 16 ^,^ 18 ^,^ 40 ^,^ 41 , the EPPM (5 studies) 19 ^,^ 20 ^,^ 21 ^,^ 25 ^,^ 47 , the TPB (4 studies) 16 ^,^ 17 ^,^ 22 ^,^ 23, and the social cognitive model (modified and applied by some studies) 27 ^,^ 28 ^,^ 30 ^,^ 35 ^,^ 36. In terms of the hazard targeted, different forms of influenza (H1N1 and H5N1) and SARS were the predominant focus (14 articles) 14 ^,^ 15 ^,^ 16 ^,^ 17 ^,^ 18 ^,^ 19 ^,^ 20 ^,^ 21 ^,^ 22 ^,^ 23 ^,^ 24 ^,^ 25 ^,^ 26 ^,^ 27 ^,^ 28, followed by flood (seven articles) 29 ^,^ 30 ^,^ 31 ^,^ 32 ^,^ 33 ^,^ 34 ^,^ 35 and earthquake (four articles) 35 ^,^ 36 ^,^ 37 ^,^ 38.

Evidence which emerged from studies applying the HBM to investigate preparedness for influenza demonstrated that perceived susceptibility, perceived severity, perceived benefits, and perceived barriers were able to predict preparedness for disease outbreaks (using logistic regression) 14 ^,^ 16 ^,^ 18. However, the study that used the same model (HBM) and the same method of analysis for preparedness for heat wave hazards showed that risk perception (perceived susceptibility and severity) and perceived barriers were not associated with preparedness 41. While it must remain tentative until more research is conducted, this raises the possibility that systematic study across hazards is required to ascertain whether hazard preparedness differ depending on the specific hazard. Another potential issue raised here concerns the relative exposure people have had to influenza versus heatwave hazards. Levels of coverage of heatwaves have, in the past, been considerably lower than that for influenza and other health hazards. This lack of coverage could affect people’s ability to personalize their risk and thus affect how they interpret issues such as perceived susceptibility and severity. Again, this idea remains speculative until more work is conducted, and this should be done before making broad decisions about the utility or otherwise of a theory.

One study used the HBM and involved both logistic regression and structural equation model analyses. In the logistic regression analysis, perceived barriers and perceived cues to action were the only elements from the HBM that demonstrated an association with preparedness for climate change. However, in structural equation model analysis, perceived susceptibility, perceived severity, perceived benefit, and perceived barrier were associated with having an emergency kit, some directly and others indirectly 40. Therefore, apart from the study settings, size, and population type (in some high risk group population selected for study) that challenge the validity of the findings of some studies, the method of analysis used to show the relationship of constructs proved to be very crucial for demonstrating the predictive utility of the theory for hazard preparedness and most studies that applied HBM did demonstrate this 14 ^,^ 15 ^,^ 16 ^,^ 18 ^,^ 41 . This issue applies to testing all theories and calls for future work on process models such as those discussed in this paper to be analyzed using at least path analysis and preferably structural equation modelling.

The second most commonly used model was the EPPM. Three of the studies conducted using this theory demonstrated, using logistic regression analysis, that higher perceived threat and higher efficacy predicted the willingness of health workers to respond to emergency when either required or asked 19 ^,^ 20 ^,^ 21 and one study reported the significant association of only self-efficacy to preparedness 47. The fourth study, which used structural equation model for the analysis, demonstrated that threat, being mediated by fear arousal, predicted behavioral intention for preparedness, while self-efficacy was directly associated with behavioral intention for preparedness 25. In five of the studies reviewed, the methods of study participants’ selection was random sampling, the sample sizes were also high in four of them and the outcomes of the studies strongly supports the applicability of EPPM in disaster and emergency preparedness (particularly when the focus is on self-efficacy and threat). However, the mediating effect of fear observed in the association of threat and preparedness, as shown in one study using path analysis 25. This reiterates the need for future work applying the EPPM to not only include self-efficacy and threat but also to expand the model further through the inclusion of other variables.

The next most commonly applied theory was the TPB. Four studies operationalized TPB to investigate disease outbreaks (influenza) related preparedness. In two of these studies, the method of analysis used was “hierarchical ordinary least square regression.” These studies reported the existence of an association between attitude and intention/getting of the influenza vaccination, and between subjective norm and intention/getting of the influenza vaccination, as part of preparedness measures; while self-efficacy was not associated with preparedness 16 ^,^ 17. However, in another study, that analyzed relationships using path analysis, a strong association of self-efficacy with intention and preparedness was reported, as well as an association between attitude and intention and preparedness 22. As described in the results (see above), the other study in this context failed to produce clear evidence of an ability to predict preparedness 23. Overall, the TPB has been shown to be one of the more influential theories applied in disaster and emergency preparedness settings. However, consistent with findings from studies using alternative theories/models, issues regarding the empirical demonstration of the the association of variables or constructs in the TPB between each other and preparedness is limited. This provides further support for the need for more rigorous path analytical or structural equation modelling (SEM) analyses of the theory across a range of hazards.

The social cognitive model was another model with a history of application across various hazards (influenza, flood, earthquake, tornado and others). It sought to investigate the influence of cognitive, affective, emotional and social factors on preparedness. The constructs that comprise the social cognitive model were modified in different studies to investigate the specific route and predictive power of the chosen variables in relation to either intention to prepare or actual preparedness. The selection of intention as a dependent variable derived from a need to provide a common dependent variable when testing the theory on hazards that differed with regard to their specific preparedness content. From the studies identified for this systematic review, the validity of the social cognitive model was supported by studies typically using path analysis or SEM to demonstrate the interdependence relationships between variables, although some studies described the direct influence of specific variables on intention or actual preparedness for emergencies and disasters 27 ^,^ 28 ^,^ 30 ^,^ 35 ^,^ 36.

Apart from the theories and models discussed above, other theories such as PMT 26, PADM 29, Precaution Adoption Theory 42, TTM 46, and others being derived from other individually focused theories and social theories had been used in disasters and emergencies preparedness. Particularly, the studies addressing the terrorism derived their models from other popular theories, and hence further re-testing in different social-cultural settings is needed 44 ^,^ 45. At times, studies had also used a combination of two theories/models, either for the purpose of comparison or to integrate their constructs for the reason of complementary to each other; for instance MLOC and HBM 18 had been compared to each other, while the HBM and TBP had been integrated 16 to see the mediating effects of the constructs of HBM on constructs of TBP to predict the intention for preparedness. However, with regard to hazard type, the natural hazards and diseases outbreaks had been more investigated with theories and models; no specific man-made hazards, apart from general terrorism, had been specifically focused.

The search strategies displayed in the methodology section evolved from the general terms such as “theory”, “model” and “disaster” to the combination of both general and specific keywords using conjunction “OR”. This was done so, because during the pilot testing, the numbers of articles generated by using only general terms from some of the databases were fewer (particularly from PubMed and Nursing Index databases) than the combination of general and specific keywords. Therefore, we decided to include the specific terminologies for behaviors/model that were familiar to us from the pilot-study exercise and our own experiences (e.g. health belief model, theory of planned behavior and etc…); disasters (natural disasters such as earthquake and flood, man-made disasters chemical and biological terrorism); and for the term preparedness alternative terminologies such as protective action, adaptive behavior, and others were incorporated into search strategies. These search strategies were able to generate studies with specified theories and models that their keywords were included, and also other studies with behavioral theories and models that their keywords were not included. For example, studies with Witte’s Extended Parallel Process Model, Vested Interest Theory, and Protective Motivation Theory (PMT) were obtained despite their specific keywords were not in search strategies. The appropriateness of the search terms used is supported by the fact that the search did identify papers covering the most commonly used theories and models and brought to light those less frequently used in the preparedness literature.Hence, while the inclusion of specific terms along with general terms found to capture more studies with theories and models even whose specific terms were unspecified in search strategies, still we do feel non-inclusion of some specific terms (for theories and models unfamiliar to us in disaster field) might have resulted in some relevant studies of our interest being omitted, and that is one of the caveats that needs to be addressed by future studies. It is also important to note that the exclusion criteria meant that several studies were identified by their use of one or more of the above terms in their title, keywords or abstract were excluded because they did not include a systematic empirical analysis of the theory or model. Future work should also be directed to exploring how and why studies did so. For example, did this occur because researchers felt that the theory was inappropriate in some way? This possibility could be explored in future studies as a way of delving deeper into the utility or otherwise of behavioral change theory approaches to hazard preparedness.

One of the limitations of this systematic review is that the articles were searched only in four databases that were accessible; other databases such as Web of Science and CINAHL were not accessed. Moreover, during the full text review of articles, six articles were not accessed and they were not included for the review. Due to different methods of statistical analysis used across the studies (logistic regression, “hierarchical ordinary least square regression”, and structural equation model) this made it difficult to summarize the results under each category of hazard type. However, efforts have been made to summarize where possible and in other instances results were presented qualitatively for the sake of clarity. Despite these limitations, we do believe that this systematic review could contribute to the existing literature on disasters and emergencies preparedness. The content provides a good foundation for future comparative studies. The results offer tentative support for the all-hazards utility of several theories. However, this review also identified a need for more systematic all-hazards testing of all these theories.

The search strategy identified only three studies from Asia. This identifies a need for additional research to target the use of behavioral change theories in the Asian countries that bear the brunt of disasters and their consequences. The importance of testing these theories in Asia derives from both the greater incidence and magnitude of disasters and their consequences in Asia and from the fact that the cultural characteristics of Asian countries could create behavior change contexts that differ significantly from those in the western countries in which the theories were developed and tested. For example, differences in the individualism-collectivism, power distance and uncertainty avoidance cultural characteristics between Asian and western countries 48 have implications for the validity of the variables in the above theories and for the preparedness process per se. Future work should include more cross-cultural and all-hazards work.

## CONCLUSIONS

Based on the articles archived and selected, behavioral theories and models are applied to disasters and emergencies preparedness more commonly in developed countries (USA and Europe). In Asia, where the annual number of disasters events and victims exceed those in other continents, only 3 studies applying behavioral theories and models to disasters and emergencies were identifies. This identified a need for additional research to target the use of behavioral change theories in the Asian countries that bear the brunt of disasters and their consequences. This does not, however, mean that these theories have not been used in Asian contexts. Our study only focused on the application of behavioral theories and models to the preparedness dimension of disaster management. Future work could expand to explore whether these theories have been used in response and recovery settings in Asia and elsewhere.

HBM, EPPM, TPB and social cognitive theories were the most commonly used for different forms of hazards preparedness. These hazards were preponderantly various forms of influenza (H1N1 and H5N1), floods, and earthquake; no specific man-made hazards were focused, apart from the general terrorism. Theoretically, models and theories have elements or constructs, which are interdependent to each other and then finally influencing the main dependent variable within constructs. Hence, studies ideally purported to reveal this interdependence of variables/elements/constructs, besides demonstrating the direct influence of some specific constructs on the main dependent variable/construct. One of the methods of analyses that could serve that purpose is structural equation model; however, most of the studies did not give the information pertaining to the influence of each construct on each other and the analyses used were traditional logistic regressions. Studies that used social cognitive theory portrayed their results in terms of path analyses, and few of the studies with other theories and models showed the relationship of constructs to each other and to the main dependent variable. Nevertheless, the theories and models applied to disasters and emergencies preparedness provided strong evidence, which could guide public health professionals, disaster management bodies and other actors in targeting interventions at preparedness phase of disaster management and emergency.

## RECOMMENDATIONS

This study is a preliminary analysis based on 1.6% of total articles generated mainly from four databases, and hence future similar systematic reviews need to build stronger sensitive search strategies and incorporate other databases that we couldn’t get access to. Moreover, future original studies related to behavioral theories and models addressing preparedness need to target developing countries where disaster risk and the consequent need for preparedness is high. This study also identified a need for additional work to demonstrate the relationships of variables and constructs, including more clearly articulating roles for mediating effects.

## COMPETING INTERESTS

Luche Tadesse Ejeta, Ali Ardalan and Douglas Paton have declared that no conflicts of interest exist.

## CORRESPONDENCE

Corresponding author: Ali Ardalan, MD, PhD. School of Public Health, International Campus, Tehran University of Medical Sciences ; Harvard Humanitarian Initiative, Harvard University.

Email: aardalan@tums.ac.ir, ardalan@hsph.harvard.edu

## APPENDIX 1

**PRISMA Checklist:** Page numbers refer to the original manuscript

Section/topic	#	Checklist item	Reported on page #
TITLE			
Title	1	Identify the report as a systematic review, meta-analysis, or both.	1
ABSTRACT	2	Provide a structured summary including, as applicable: background; objectives; data sources; study eligibility criteria, participants, and interventions; study appraisal and synthesis methods; results; limitations; conclusions and implications of key findings; systematic review registration number.	1
INTRODUCTION			
Rational	3	Describe the rationale for the review in the context of what is already known.	3
Objectives	4	Provide an explicit statement of questions being addressed with reference to participants, interventions, comparisons, outcomes, and study design (PICOS).	3
METHODS			
Protocol and registration	5	Indicate if a review protocol exists, if and where it can be accessed (e.g., Web address), and, if available, provide registration information including registration number.	N/A
Eligibility criteria	6	Specify study characteristics (e.g., PICOS, length of follow-up) and report characteristics (e.g., years considered, language, publication status) used as criteria for eligibility, giving rationale.	4
Information sources	7	Describe all information sources (e.g., databases with dates of coverage, contact with study authors to identify additional studies) in the search and date last searched.	4-5
Search	8	Present full electronic search strategy for at least one database, including any limits used, such that it could be repeated.	4-5
Study selection	9	State the process for selecting studies (i.e., screening, eligibility, included in systematic review, and, if applicable, included in the meta-analysis).	6
Data collection process	10	Describe method of data extraction from reports (e.g., piloted forms, independently, in duplicate) and any processes for obtaining and confirming data from investigators.	6
Data items	11	List and define all variables for which data were sought (e.g., PICOS, funding sources) and any assumptions and simplifications made.	6
Risk of bias in individual studies	12	Describe methods used for assessing risk of bias of individual studies (including specification of whether this was done at the study or outcome level), and how this information is to be used in any data synthesis.	6
Summary measures	13	State the principal summary measures (e.g., risk ratio, difference in means).	N/A
Synthesis of results	14	Describe the methods of handling data and combining results of studies, if done, including measures of consistency (e.g., I2) for each meta-analysis.	N/A
Risk of bias across studies	15	Specify any assessment of risk of bias that may affect the cumulative evidence (e.g., publication bias, selective reporting within studies).	N/A
Additional analyses	16	Describe methods of additional analyses (e.g., sensitivity or subgroup analyses, meta-regression), if done, indicating which were pre-specified.	N/A
RESULTS			
Study selection	17	Give numbers of studies screened, assessed for eligibility, and included in the review, with reasons for exclusions at each stage, ideally with a flow diagram.	6-7
Study characteristics	18	For each study, present characteristics for which data were extracted (e.g., study size, PICOS, follow-up period) and provide the citations.	8-17 and Appendix 2
Risk of bias within studies	19	Present data on risk of bias of each study and, if available, any outcome level assessment (see item 12).	8-17
Results of individual studies	20	For all outcomes considered (benefits or harms), present, for each study: (a) simple summary data for each intervention group (b) effect estimates and confidence intervals, ideally with a forest plot.	8-17
Synthesis of results	21	Present results of each meta-analysis done, including confidence intervals and measures of consistency.	N/A
Risk of bias across studies	22	Present results of any assessment of risk of bias across studies (see Item 15).	8-17
Additional analysis	23	Give results of additional analyses, if done (e.g., sensitivity or subgroup analyses, meta-regression [see Item 16]).	N/A
DISCUSSION			
Summary of evidence	24	Summarize the main findings including the strength of evidence for each main outcome; consider their relevance to key groups (e.g., healthcare providers, users, and policy makers).	18-20
Limitations	25	Discuss limitations at study and outcome level (e.g., risk of bias), and at review-level (e.g., incomplete retrieval of identified research, reporting bias).	20
Conclusions	26	Provide a general interpretation of the results in the context of other evidence, and implications for future research.	21
FUNDING			
Funding	27	Describe sources of funding for the systematic review and other support (e.g., supply of data); role of funders for the systematic review.	21

N/A: Not Applicable

From: Moher D, Liberati A, Tetzlaff J, Altman DG, The PRISMA Group (2009). Preferred Reporting Items for Systematic Reviews and Meta-Analyses: The PRISMA Statement. PLoS Med 6(6): e1000097. doi:10.1371/journal.pmed1000097

## APPENDIX 2

**Summary of studies and characteristics**

**Figure:**
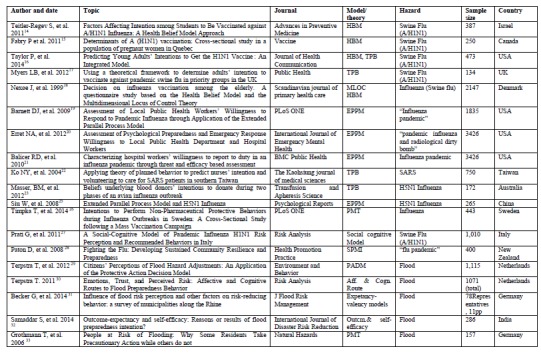
**Appendix 2. Table 1**

**Figure:**
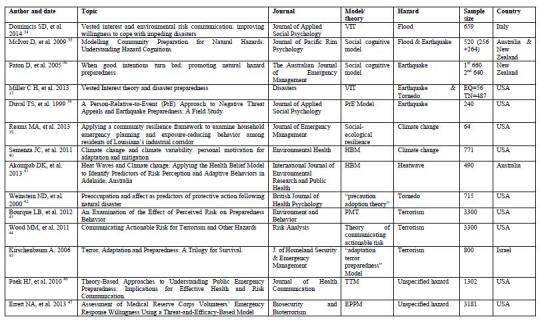
**Appendix 2. Table 2**
